# Supplementary material for: Chronological aging impacts abundance, function and microRNA content of extracellular vesicles produced by human epidermal keratinocytes
Source: Aging (Albany NY). 2023 Nov 27;15(22):12702–22. doi: 10.18632/aging.205245 (PMC10713413; doi:10.18632/aging.205245)
Supplement: Supplementary Figures [file aging-15-205245-s001.pdf]

## SUPPLEMENTARY FIGURES

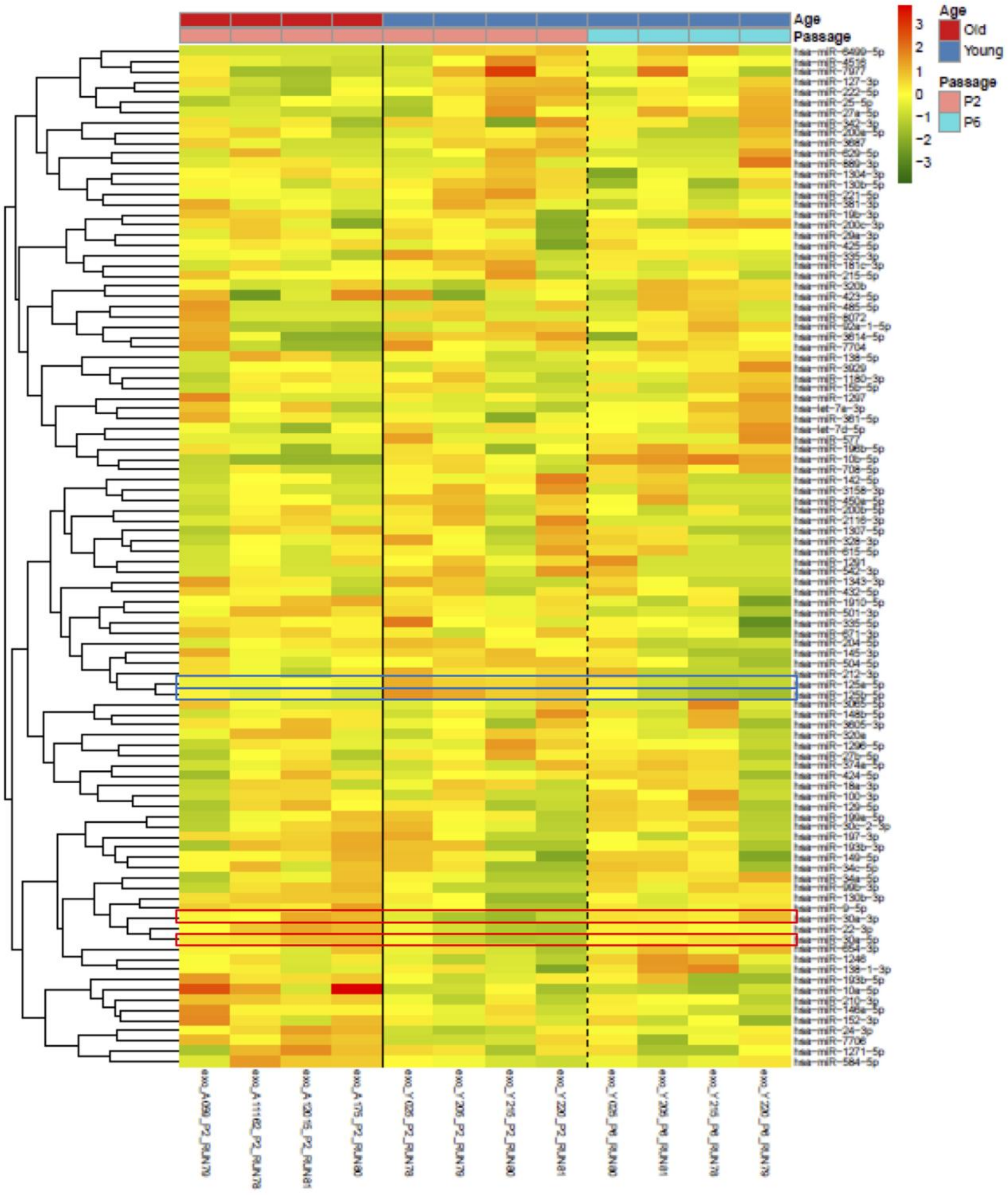


**Supplementary Figure 1. Heatmap representing the expression of the 100 miRNAs with the highest variance in the 12 EV samples analyzed in our study.** The miRNAs that are significantly (*P*adj < 0.05) induced in old keratinocytes EVs compared to young keratinocytes EVs at early passage (P2) are indicated by a red box. The miRNAs that are significantly (*P*adj < 0.05) repressed in old keratinocytes EVs compared to young keratinocytes EVs at early passage (P2) are indicated by a blue box.

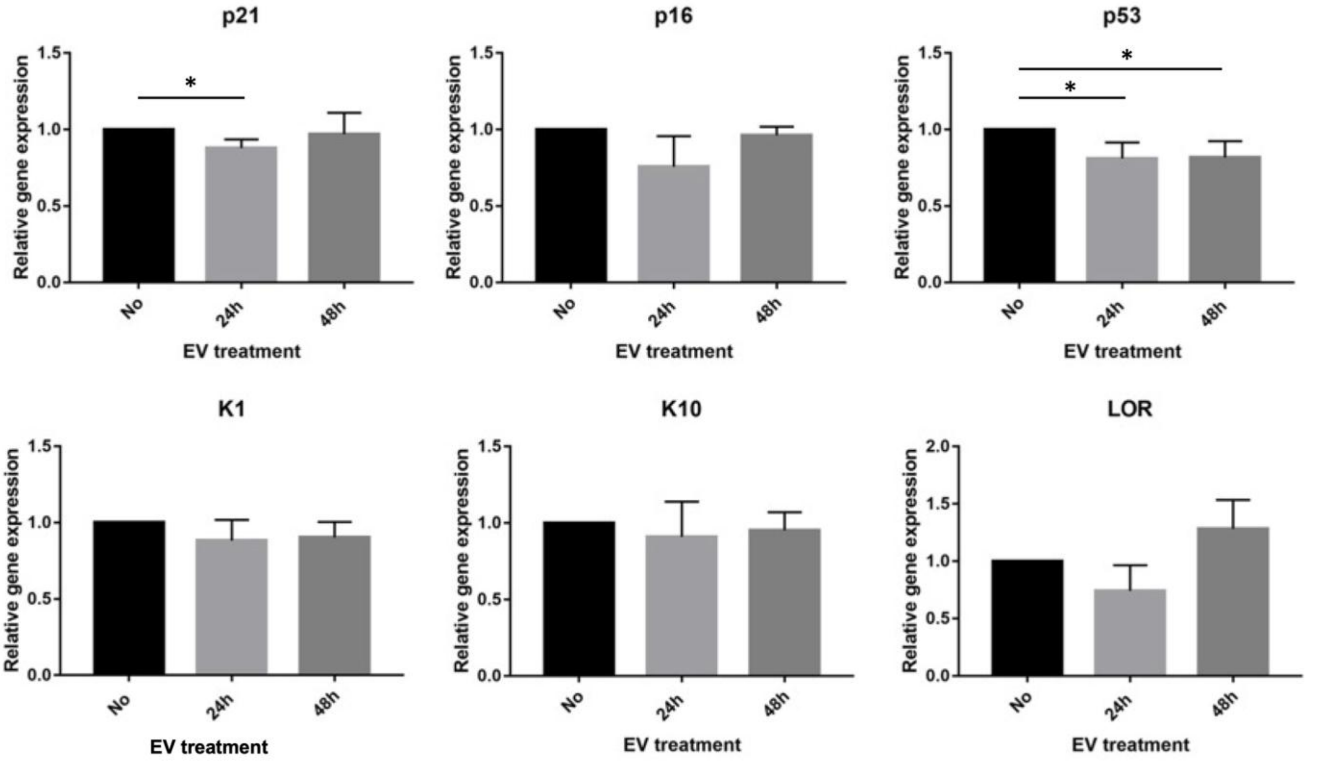


**Supplementary Figure 2. Expression analysis of transcripts for P21, P16, P53, KRT1 (K1), KRT10 (K10) and loricrin (LOR) in young keratinocytes treated with EVs from young keratinocytes.** Data were normalized to the non-treated condition. (*$p$-value < 0.05. $n$ = 3 Student's $t$-test).

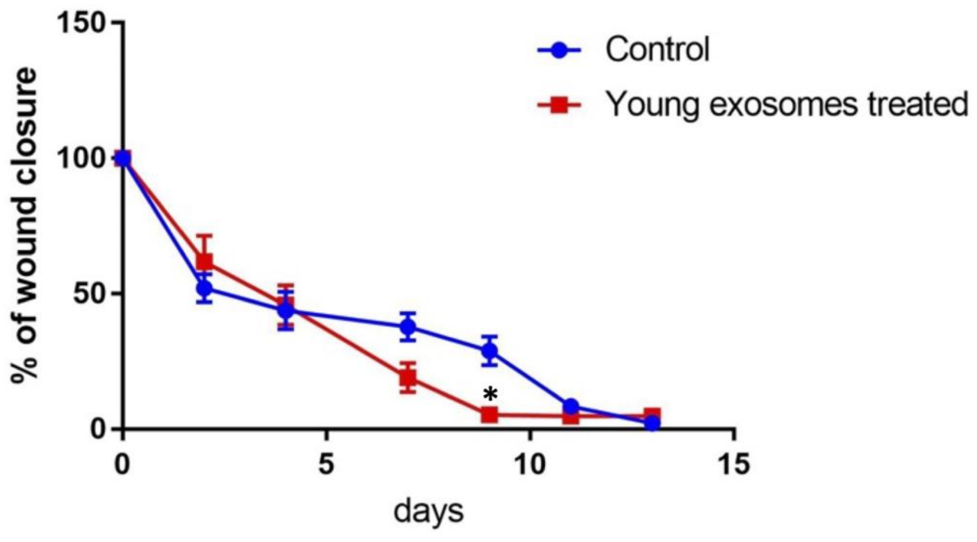


**Supplementary Figure 3. Wound closure assay on mice treated with EVs from young keratinocytes.** Quantitative analysis of wound healing closure in each group ($n$ = 4), expressed as a percentage of the initial wound area (*$p$-value < 0.05. Student's $t$-test).
